# Supplementary figures and images for: Mmp17-deficient mice exhibit heightened goblet cell effector expression in the colon and increased resistance to chronic Trichuris muris infection
Source: Front Immunol. 2023 Oct 6;14:1243528. doi: 10.3389/fimmu.2023.1243528 (PMC10587605; doi:10.3389/fimmu.2023.1243528)

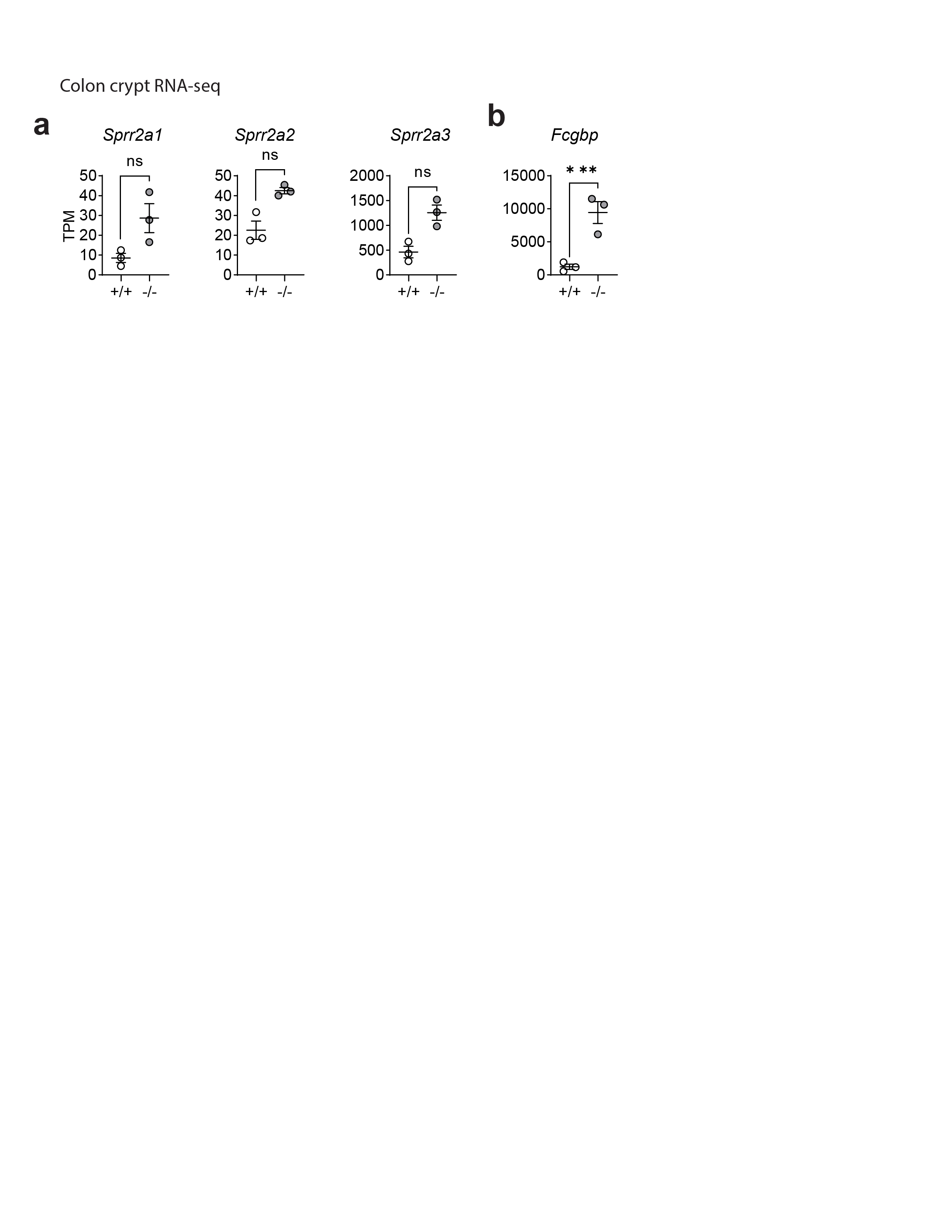

Supplement: Supplementary Figure 1 — MMP17 controls goblet cell gene expression in Colon. (A) RNAseq data of naive mouse colon crypts from WT (+/+) and Mmp17 KO (-/-) mice showing the expression of for Sprr2a1, Sprr2a2, Sprr2a3 and Fcgbp (B), n=3, pa dj * p<0.05, ** p<0.01, *** p<0.00. Numerical data are means ± SEM. Data represents p-adjusted value from RNAseq analysis. [file Image_1.jpeg]

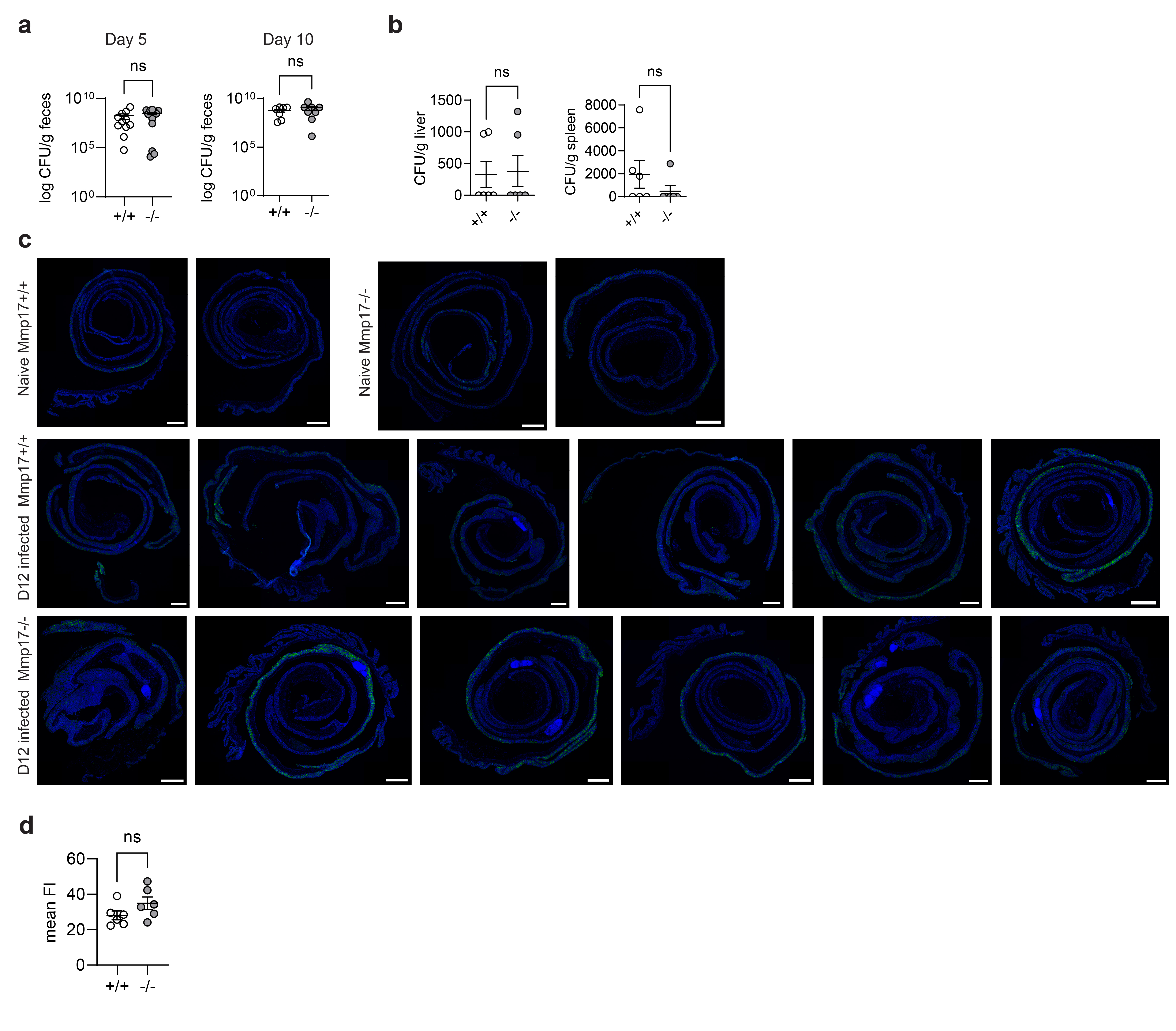

Supplement: Supplementary Figure 2 — C. rodentium infection of Mmp17 WT and KO mice. (A) CFU per gram feces at day 5 (left) and day 10 (right) post infection; n(day 5)=14; n(day 10)=8. (B) CFU per gram of homogenized liver (left) and spleen (right) at day 12 post infection; n=6. (C) Immunofluorescence (IF) images of Colon Swiss rolls of naïve and C. rodentium infected mouse full-length colon of WT (+/+) and Mmp17 KO (-/-) mice, for RELM-β (green), nuclear stain (blue), scale 1 mm. (D) Mean fluorescence intensity in proximal colon crypts in C. rodentium infected mice; n=6. [file Image_2.jpeg]

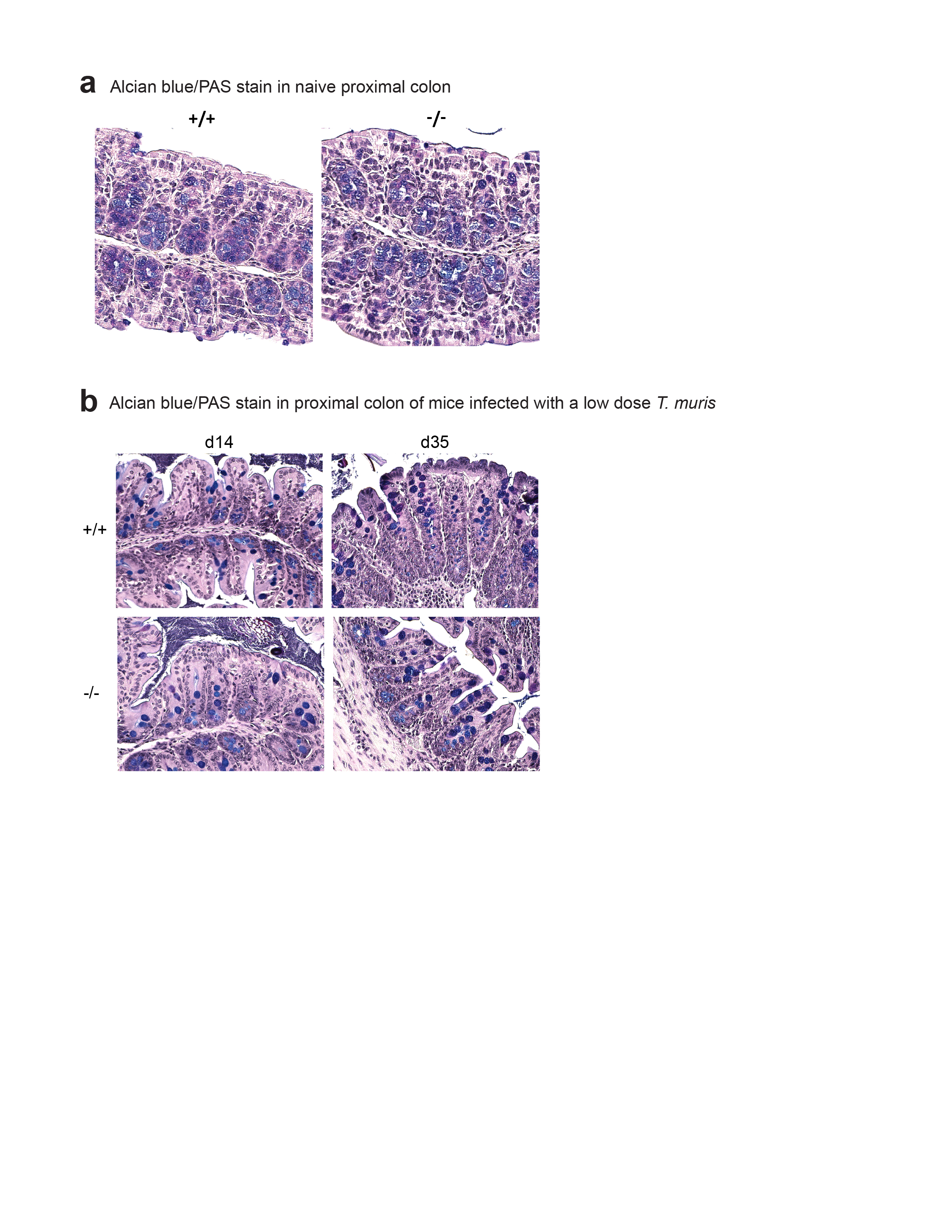

Supplement: Supplementary Figure 3 — Alcian blue/PAS stain of naive mouse proximal colon of WT (+/+) and Mmp17 KO (-/-) mice at 14 dpi and 35 dpi. [file Image_3.jpeg]
